# Supplementary material for: Survey of awareness of radiation disasters among firefighters in a Japanese prefecture without nuclear power plants
Source: PLoS One. 2020 Jul 27;15(7):e0236640. doi: 10.1371/journal.pone.0236640 (PMC7384620; doi:10.1371/journal.pone.0236640)
Supplement: S1 File — (DOCX) [file pone.0236640.s001.docx]

**Supplementary materials**

The detailed contents of the questionnaire were as follows.

The characteristics of the participants:

(i) age: under 30 (<30), 30–39 (30s), 40–49 (40s), 50–59 (50s), and 60 and over (≥60);

(ii) marital status: married or unmarried;

(iii) duration of service: less than six years; 6–10 years; 11–20 years; 21–30 years; 31–40 years; and over 40 years;

(iv) details of duties: routine duties (administration), fire suppression duties, rescue duties, first-aid duties, and command duties (Table S1);

**Table S1. Details of Tasks.**

| **Name of task** | **Details** |  |
| --- | --- | --- |
| Fire suppression | Responding to fires, floods, earthquakes, and other disasters. |  |
| First aid | Treating injured people at the disaster site and transporting them to hospital. |  |
| Rescue | Rescuing people at the disaster site. |  |
| Communication | Handling 119 telephone calls and communicated instructions. |  |
| Prevention  (Routine tasks) | Instructions about assessment of buildings and dangerous items and overall fire department administration. |  |

(v) experience of mobilisation to large-scale disasters and amount of such mobilisations;

(vi) experience of mobilisation to radiation disasters and amount of such mobilisations.

The items relating to awareness about the effects of nuclear incidents:

(i) interest in events at the time of nuclear incidents: ‘very interested’, ‘interested’, ‘not really interested’, or ‘not at all interested’;

(ii) awareness of the effects of nuclear incidents on participants’ own health: ‘I think there are effects’, ‘I do not think there are any effects’, or ‘I have not thought about it’;

(iii) awareness of the effects of nuclear incidents on the health of adults and children: ‘I think there are effects’, ‘If I have to choose, I think there are effects’, ‘If I have to choose, I think there are no effects’, or ‘I think there are no effects’;

(iv) awareness of genetic effects: ‘I think there are effects’, ‘If I have to choose, I think there are effects’, ‘If I have to choose, I think there are no effects’, or ‘I think there are no effects’.

The items relating to awareness about radiation disasters:

(i) potential for a nuclear disaster in the area where one is stationed: ‘I think it is possible’ or ‘I do not think it is possible’;

(ii) level of confidence in responding to radiation disaster: ‘confident’, ‘anxious but okay’, or ‘not confident’;

(iii) understanding of the details of firefighting teams’ activities at the time of the Fukushima Daiichi Disaster: ‘detailed’, ‘basic’, or ‘none’;

(iv) experience of attending lectures about radiation disasters and/or emergency exposure situations: ‘before the Fukushima Daiichi Disaster’, ‘after the Fukushima Daiichi Disaster’, ‘both before and after the Fukushima Daiichi Disaster’, or ‘none’;

(v) participants’ own competence at using the materials and/or equipment for radiation disasters owned by the fire department: ‘I can use them’, ‘I understand the details and location of the materials and/or equipment’, or ‘I cannot use them’;

(vi) understanding of the *Manual for Firefighting Activities at Nuclear Power Facilities, Etc.* released by the Ministry of Internal Affairs and Communications’ Fire and Disaster Management Agency in March 2014: ‘full understanding’, ‘partial understanding’, or ‘no understanding’;

(vii) wish to participate in future seminars about radiation disasters: ‘I will definitely participate’, ‘I would be willing to participate’, ‘I would perhaps participate’, or ‘I would not participate’;

(viii) reasons for not participating in seminars about radiation disasters: ‘I have already undergone the required education and training’, ‘radiation disasters do not occur frequently’, ‘I have numerous other things to do’, ‘I do not think participating would have any positive results’, or ‘other’;

(ix) participants’ response if voluntary transfer to a team that is mobilised preferentially for radiation disasters is proposed: ‘I would accept without hesitation’, ‘I would accept under certain conditions’, or ‘I would refuse’;

(x) conditions under which one would accept the transfer: ‘I would be provided with sufficient education and training before assignment’, ‘Education and training would continue after assignment’, ‘I would have satisfactory personal equipment and health checks’, ‘The pay would be very good’, or ‘others’;

(xi) reasons for rejecting such a transfer: ‘Working in a radiation disaster would be dangerous’, ‘I do not want to be subjected to more training’, ‘Taking my family into consideration, I would be anxious’, ‘I think it would be best for people to willingly take up such work’, or ‘others’;

(xii) views about voluntarily working in an area where the air radiation dose rate has been measured to be 100 mSv/year, if this is proposed: ‘I would participate’ or ‘I would refuse’;

(xiii) reasons for participating in such activities: ‘I am a firefighter’, ‘100 mSv/year has no effect on health’, ‘I do not think I can refuse, even if it is only a proposal’, or ‘others’;

(xiv) reasons for refusing to participate in such activities: ‘100 mSv/year will have effects on health’, ‘Taking my family into consideration, I want to avoid radiation exposure’, ‘I would be anxious because I have no experience with radiation disasters’, or ‘others’;

(xv) participants’ own preparedness for future radiation disasters: ‘I am sufficiently prepared’, ‘I cannot say that I am sufficiently prepared’, or ‘I am not sufficiently prepared’;

(xvi) reasons for a response of ‘I cannot say that I am sufficiently prepared’ or ‘I am not sufficiently prepared’ to the last question: ‘the participant’s own knowledge and education’, ‘personal equipment’, ‘understanding of the manual’, ‘self-care after completion of dispatch’, or ‘others’.
