# Supplementary material for: Survey of awareness of radiation disasters among firefighters in a Japanese prefecture without nuclear power plants
Source: PLoS One. 2020 Jul 27;15(7):e0236640. doi: 10.1371/journal.pone.0236640 (PMC7384620; doi:10.1371/journal.pone.0236640)
Supplement: Attachment — Submitted filename: Response to reviewers.doc [file pone.0236640.s002.doc]

June 5, 2020

Dear Reviewers,

Thank you for inviting us to submit a revised draft of our manuscript entitled, ‘Survey of awareness of radiation disasters among firefighters in a Japanese prefecture without nuclear power plants’ [ms. no PONE-D-19-32209] to *PLoS ONE*. We also appreciate the time and effort you and each of the reviewers have dedicated to providing insightful feedback on ways to strengthen our paper. Thus, it is with great pleasure that we resubmit our article for further consideration. We have incorporated changes that reflect the detailed suggestions you have graciously provided. We also hope that our edits and the responses we provide below satisfactorily address all the issues and concerns you and the reviewers have noted.

To facilitate your review of our revisions, the following is a point-by-point response to the questions and comments delivered in your letter dated 24 April 2020.

- **Reviewer #1:**

We are grateful to reviewer #1 for the critical comments and useful suggestions that have helped us to improve our paper considerably. As indicated in the responses that follow, we have taken all these comments and suggestions into account in the revised version of our paper.

**Comment 1.** ‘The authors conducted an important research to facilitate the understanding of the factors affecting individual’s attitudes/decisions towards radiation disaster response. I believe the study has broad impacts, and the results could help develop better disaster preparedness and response. However, the discussion about the intellectual merit of the paper is not enough. How the study would contribute to the existing knowledge of the factors affecting individual’s attitudes/decisions towards disaster preparedness and response is not fully discussed in the paper. I would urge the authors conduct a comprehensive literature review and elaborate the intellectual merits of the paper in both Introduction and Discussion sections’.

**Response.**

We have included new references about individual attitudes/decisions in the Discussion section (pp. 20-21). Furthermore, we have added discussed on how the study contributes to existing knowledge by adding more background information and new sources to the Introduction section (pp. 7-8).

**Comment 2.** ‘From the lines 145 to 210, the authors explain the survey in detail, and I feel difficult to follow here. The content is helpful for readers to understand the research but also makes the paper tedious. Maybe the authors could find a good way to put it together. For example, the authors could stress some important points in the paper and put more detailed information in the supplemental document’.

**Response.**

We have rewritten the lines to be more in line with your comments (new lines 227-251) and have provided detailed information about the questionnaire as supplementary material. We hope that the edited section is clearer and more readable.

**Comment 3.** ‘For the chi-square and ordinal logistic regression tests, I would urge the authors either inform the null hypothesis and the alternative hypothesis in the context of study or elaborate what do the results of tests interpret’.

**Response.**

We have elaborated the interpretation of the results of both tests in the Results section.

**Comment 4.** ‘The discussion related to the results is not sufficient. Also, there are no clear conclusions relevant to the emerging literature. I would urge the authors to revisit some of the literature regarding individual’s attitudes/preferences towards policies of disaster planning and response and discuss how the results could contribute to the existing literature’.

**Response.**

We have included new references about individual attitudes/decisions to the Discussion section (pp. 20-21). We also added references to literature ‘regarding individual’s attitudes/preferences towards policies of disaster planning and response’ to the Introduction (pp. 7-8). Although we could not find any sources about firefighters’ preferences, there are a few reports about medical stuff such as nurses (p. 8). Therefore, we believe that our paper’s significance lies in the fact that our study population, firefighters, has apparently been overlooked by research and also that it provides useful support to making activities safe and efficient.

**Comment 5.** ‘I suggest that the authors ask for a professional copy editing service. The paper could be greatly improved in terms of the language such as the use of voice, tense, article and the word choice. Also, a common problem is that some sentences are really long and become difficult to follow. The authors could break the long sentences down into several short sentences. I listed some examples that could be improved’.

**Response.**

We have revised the entire paper and have shortened sentences to reflect your comments. We have also had the manuscript reviewed and revised by a professional English copy editing service.

- **Reviewer #2:**

We are grateful to reviewer #2 for the critical comments and useful suggestions that have helped us to improve our paper considerably. As indicated in the responses that follow, we have taken all these comments and suggestions into account in the revised version of our paper.

**Comment 1.** ‘Profile of participants

In this paper, firefighters are classified into 5 categories. Are they engaged in general affairs? I think firefighting department may have the section responsible for special disaster for such as nuclear materials, chemical, biohazards. In this survey, descriptions about firefighters engaged in such works are needed’.

**Response.**

We have added the profiles of participants and their engagement in special disasters in the Materials and Methods section.

**Comment 2.** ‘Significance of survey in Nagasaki “without nuclear plants”

Authors describe Nagasaki as prefecture without nuclear plants. However Nagasaki severely suffered from atomic bomb in 1945. Many people in Nagasaki get intensive education from primary schools. In this point, Nagasaki is different from other prefectures in Japan. Some discussions about this point may be needed’.

**Response.**

We have added discussion of Nagasaki Prefecture’s current status and the history of the Nagasaki City Fire Department in the Discussion section. We have also mentioned the considerable influence of the bombing in this section.

With these changes to our final manuscript, we hereby resubmit our manuscript for a secondary evaluation. We hope that the revised version of our paper is now suitable for publication in *PLoS ONE*, and we look forward to hearing from you at your earliest convenience.

Sincerely,

Naomi Hayashida, M.D., PhD.

Professor of Division of Strategic Collaborative Research, Center for Promotion of Collaborative Research on Radiation and Environment Health Effects, Atomic Bomb Disease Institute, Nagasaki University

1-12-4 Sakamoto, Nagasaki 852-8523, JAPAN

TEL: +81-95-819-8507

FAX: +81-95-819-8508

E-mail: [naomin@nagasaki-u.ac.jp](mailto:naomin@nagasaki-u.ac.jp)
